# The legume miR1514a modulates a NAC transcription factor transcript to trigger phasiRNA formation in response to drought

**DOI:** 10.1093/jxb/erw380

**Published:** 2016-10-07

**Authors:** Guadalupe Sosa-Valencia, Miguel Palomar, Alejandra A. Covarrubias, José L. Reyes

**Affiliations:** 1 Departamento de Biología Molecular de Plantas, Instituto de Biotecnología, Universidad Nacional Autónoma de México,Av. Universidad 2001, Col. Chamilpa, C.P. 62210, Cuernavaca Mor., Mexico

**Keywords:** ARGONAUTE 1, drought, microRNAs, phased siRNAs, *Phaseolus vulgaris*, PTGS, transcriptome.

## Abstract

Recent studies have identified microRNAs as post-transcriptional regulators involved in stress responses. miR1514a is a legume microRNA that is induced in response to drought stress in *Phaseolus vulgaris* (common bean) and shows differential accumulation levels in roots during water deficit in two cultivars with different drought tolerance phenotypes. A recent degradome analysis revealed that miR1514a targets the transcripts of two NAC transcription factors (TFs), Phvul.010g121000 and Phvul.010g120700. Furthermore, expression studies and small RNA-seq data indicate that only Phvul.010g120700 generates phasiRNAs, which also accumulate under water deficit conditions. To confirm these results, we over-expressed miR1514a in transgenic *hairy roots*, and observed a reduced accumulation of Phvul.010g120700 and an increase in NAC-derived phasiRNAs; inhibition of miR1514a activity resulted in the opposite effect. Moreover, we determined that a NAC-derived phasiRNA associates with ARGONAUTE 1 (AGO1), suggesting that it is functional. In addition, a transcriptome analysis of transgenic *hairy roots* with reduced miR1514a levels revealed several differentially expressed transcripts, mainly involved in metabolism and stress responses, suggesting they are regulated by the NAC TF and/or by phasiRNAs. This work therefore demonstrates the participation of miR1514 in the regulation of a NAC transcription factor transcript through phasiRNA production during the plant response to water deficit.

## Highlight

*Phaseolus vulgaris* miR1514a, a non-conserved microRNA that is induced by drought, targets a NAC transcription factor transcript for degradation, consequently triggering phasiRNA production and affecting downstream genes, thus revealing their involvement in responses to drought.

## Introduction

Plants are constantly exposed to adverse conditions, such as limited water availability, that affect their growth, productivity, and development (Buchanan. *et al.*, 2015). To contend with water deficit, plants have developed diverse strategies at the molecular, cellular, and physiological levels, thus increasing their plasticity and consequently their probability of survival (Bray, 1997; Oliver *et al.*, 2010). The Fabaceae family (Legumes) includes 18 000 described species within approximately 700 genera, and they represent one-third of the primary crop production in the world; however, legume production necessary for feed and food relies on only a few cultivated species (Doyle and Luckow, 2003). Common bean (*Phaseolus vulgaris* L.) is the most important legume for human consumption and serves as a primary source of protein in the human diet in Central and South America (Broughton *et al.*, 2003; Graham and Vance, 2003; Miklas *et al.*, 2006; Castro-Guerrero *et al.*, 2016). However, the lack of proper irrigation systems and the ensuing exposure to significant drought periods impact directly on the yields of common bean in most areas where it is produced, affecting around 60% of its production. For these reasons, many breeding programs have focused on obtaining drought-resistant cultivars (Gallegos and Shibata, 1989; Broughton *et al.*, 2003). The characterization of stress responses at the molecular level among existing germplasm might reveal mechanisms underlying resistance and tolerance to drought stress.

MicroRNAs (miRNAs) are a class of non- coding small RNAs of 21–24 nucleotides (nt) in length with regulatory roles in gene expression at the post-transcriptional level through mRNA cleavage or translation repression. In plants, miRNAs are involved in the control of diverse processes such as phytohormone regulation, morphogenesis, development, and stress responses. Most knowledge of post-transcriptional gene silencing (PTGS) mediated by miRNAs in response to stress has been gained in *Arabidopsis thaliana* and a few other model species (Mallory and Vaucheret, 2006; Voinnet, 2009; Covarrubias and Reyes, 2010; Sunkar, 2010; Sosa-Valencia *et al.*, 2013), despite an apparent close co-evolution between environment adaptation and gene-silencing mechanisms mediated by miRNAs being widespread in plants (Borges and Martienssen, 2015).

In the particular case of common bean, genome-wide studies have reported more than a hundred miRNAs encoded in its genome (Pelaez *et al.*, 2012; Formey *et al.*, 2015). A few studies have described common bean miRNAs responding to different stress factors such as nutrient deficit, metal toxicity, salinity, phosphorous deficiency, and copper homeostasis (Valdes-Lopez *et al.*, 2008, 2010; Naya *et al.*, 2014; Nova-Franco *et al.*, 2015). However, miRNA involvement in drought responses of common been remains largely unexplored (Arenas-Huertero *et al.*, 2009; Contreras-Cubas *et al.*, 2012).

miRNA activities in plants commonly result in cleavage of the target transcript followed by its degradation. One alternative pathway in miRNA function in plants results from the cleavage and the recruitment of the cleaved RNA fragment by SUPPRESSOR OF GENE SILENCING 3 (SGS3) and RNA DEPENDENT RNA POLYMERASE 6 (RDR6) for double-strand RNA (dsRNA) formation and its subsequent cleavage by Dicer-like 4 (DCL4) at consecutive 21-nt intervals. This process results in the production of phased small RNAs, which were initially named *trans*-acting siRNAs (tasiRNAs) because they caused down-regulation of their own target mRNAs *in trans* (Vazquez *et al.*, 2004). Not all phased siRNAs are known to act *in trans* and therefore, more recently, they have been renamed as phased secondary siRNAs (phasiRNAs) (Jeong *et al.*, 2011; Fei *et al.*, 2013).

The phasiRNA mechanism is well described for model plants such as *Arabidopsis thaliana*, which contains a few well-characterized *PHAS* loci (Vazquez *et al.*, 2004; Fahlgren *et al.*, 2007). In contrast, little is known for other plant species commonly possessing a larger number of uncharacterized *PHAS* loci, such as *Vitis vinifera* (grape) or *Populus trichocarpa* (poplar) (Zheng *et al.*, 2015). In particular, phasiRNAs can be produced by members of the *PPR*, *NB-LRR*, and *MYB* gene families in Arabidopsis, *Medicago truncatula*, *Malus domestica*, and *Prunus persica* (Fahlgren *et al.*, 2007; Jeong *et al.*, 2011; Xia *et al.*, 2012; Zhu *et al.*, 2012). More recently, a catalog of *PHAS* loci has been described for *P. vulgaris* (Formey *et al.*, 2015), but their involvement in water deficit responses remains unknown.

The aim of this work was to contribute to the knowledge of post-transcriptional regulation in common bean mediated by the legume-specific miR1514a induced during drought stress (Arenas-Huertero *et al.*, 2009). Our analyses demonstrated that miR1514a targets the transcript encoding Phvul.010g120700 (hereafter named NAC 700), a member of the family known as No Apical Meristem (NAM) or NAC family of transcription factors (Olsen *et al.*, 2005), through cleavage and subsequent generation of secondary phasiRNAs. Here, we show that this process occurs during the exposure of adult plants to drought. Notably, we found that one resulting phasiRNA is recruited to ARGONAUTE 1- (AGO1-) complexes, supporting its role in PTGS. Furthermore, based on an RNA-seq strategy we propose downstream regulatory targets of the transcription factor NAC 700.

## Materials and methods

### Plant material and growth conditions

We used *Phaseolus vulgaris* L. (common bean) adult and seedling roots of two Mesoamerican cultivars (kindly provided by Dr Jorge Acosta of INIFAP): ‘Bayo Madero’ (drought-susceptible) and ‘Pinto Saltillo’ (drought-resistant) as described previously (Acosta-Gallegos *et al.*, 1999; Terán and Singh, 2002). For experiments with adult plants we used the biological material reported in Rosales *et al.* (2012). Plants were grown under greenhouse conditions, at 24 ± 4 °C with a relative humidity of 70 ± 20%, and a natural light/dark cycle. At 29 d after germination (DAG), 252 plants were divided in three groups and subjected to three irrigation treatments: optimal irrigation, where pots were irrigated up to field capacity (fc, 2.5 ml g^−1^ substrate); moderate drought (1.25 ml g^−1^ substrate); and severe drought (0.625 ml g^−1^ substrate) (Cuellar-Ortiz *et al.*, 2008). Leaves and roots were collected at 13 and 22 d of treatment and stored at –70 °C until further use.

To obtain seedlings, common bean seeds of the Pinto Saltillo and Bayo Madero cultivars were surface-sterilized using commercial alcohol and a bleach solution and germinated for 5 d at 26–28 °C and 60% relative humidity (RH) in darkness. They were then transplanted to vermiculite irrigated with a nutrient solution (CaCl_2_ 0.78 M, MgSO_4_ 0.486 M, ferric citrate 0.02 M, KH_2_PO_4_ 0.74 M pH 7.3, Na_2_HPO_4_ 0.84 M pH 7.3, KCl 50 mM, H_3_BO_3_ 25 mM, MnSO_4_ 5 mM, CuSO_4_ 0.5 mM, ZnSO_4_ 0.2 mM, Na_2_MoO_4_ 1 mM). For control conditions, seedlings were sown in vermiculite containing nutrient solution to 1.7 ml g^−1^ substrate. The water deficit treatment consisted of reducing the nutrient solution content to 0.425 ml g^−1^ (1/4 or mild drought) or 0.212 ml g^−1^ substrate (1/8 or severe drought), as described previously (Arenas-Huertero *et al.*, 2009). We harvested seedling hypocotyls or roots at 6, 12, 24 or 48 h after applying the water deficit conditions and they were stored at –70 °C until further use. These treatments were performed in three independent experiments.

For other treatments, ABA (65 μM), NaCl (100 and 200 mM) or Paraquat (10 and 100 μM) were added into the nutrient solution used to irrigate the seedlings sown in vermiculite as described above, and hypocotyls and seedling roots were harvested at 6, 12, 24 or 48 h after applying each treatment.

### RNA isolation and northern blot analysis

Total RNA was isolated from 100–500 mg of frozen tissue using Trizol reagent (Invitrogen, Carlsbad, CA) following the manufacturer’s instructions. Northern blot analyses were carried out as described previously (Arenas-Huertero *et al*, 2009). Synthetic DNA oligonucleotides (Unidad de Síntesis y Secuenciación de ADN, IBT-UNAM) with the antisense sequence corresponding to miR1514a and to U6 snRNA were used as probes (see Supplementary Table S5 at *JXB* online for oligonucleotide sequences; a full list of all primers used in this study is given in Table S5). Normalized miR1514a expression levels were calculated relative to U6 snRNA, as previously reported (Contreras-Cubas *et al.*, 2012; Naya *et al.*, 2014).

### Real-time quantitative RT-PCR analysis

For the quantification of miR1514a and phasiRNAs accumulation levels by RT-qPCR, cDNA was synthesized from 1 μg total RNA using the NCode miRNA First-Strand cDNA Synthesis Kit (Invitrogen) or, alternatively, using the stem-loop protocol as previously reported (Varkonyi-Gasic *et al.*, 2007) with minor adjustments as indicated by Contreras-Cubas *et al.* (2013). For qPCR we used a Step One real-time thermocycler (Applied Biosystems) and the SYBR Green PCR Master Mix (Fermentas). We performed an initial denaturing step at 95 °C for 5 min, followed by 95 °C for 10 s, 55 °C for 20 s for 40 cycles, and a final step of increasing temperature by 0.2 °C from 60 °C to 95 °C. Relative expression for each sample was calculated with the comparative C_t_ method. The C_t_ values obtained were normalized with the C_t_ value of U6 snRNA amplification.

To quantify mRNA levels of selected genes we generated cDNA using a RevertAid H Minus First Strand cDNA Synthesis Kit (Fermentas). cDNA synthesis and quality verification for RT-qPCR were performed as previously reported (Contreras-Cubas *et al.*, 2012). The resulting cDNA was then diluted and used to perform RT-qPCR assays using SYBR Green PCR Master Mix (Fermentas). Reactions were analyzed in a Step One real-time thermocycler (Applied Biosystems) using the following conditions: one cycle of 95 °C for 5 min, followed by 40 cycles of 95 °C for 30 s; 55 °C for 30 s, and extension steps were performed at specific temperatures for specific sequences. For each of three biological replicates, at least three RT-qPCR reactions were performed. Relative expression for each transcript was calculated with the comparative C_t_ method. The C_t_ values obtained were normalized with the C_t_ value of the reference gene *Skip16* (Borges *et al.*, 2012). The sequences of oligonucleotide primers used for RT-qPCR amplification of each RNA are provided in Supplementary Table S5.

Statistical analysis of gene expression (small RNAs and transcripts) was performed using one-way ANOVA and multiple paired Student’s *t*-tests.

### Immunoprecipitation of AGO1

For immunoprecipitation assays of AGO1, we used a custom-made rabbit polyclonal antibody (Genscript, Piscataway, NJ) raised against a synthetic peptide (CFYMEPETSDSGSMA) derived from AGO1 (Phvul.004G142900). The peptide sequence is located at the end of the PIWI domain at the C-terminus of the protein, and it is shared with *Arabidopsis thaliana* AGO1 and is not present in any other AGO protein encoded in the *P. vulgaris* or *A. thaliana* genomes. The antibody recognizes a single ~120 kDa protein in western blot assays. For each assay, we required 600 mg of root tissue from adult *P. vulgaris* plants ground in liquid nitrogen. A 1:50 antibody dilution was used and protein extracts were incubated in extraction buffer [50 mM Tris-HCl pH 7.5; 1.5 mM NaCl; 0.1% Nonidet 40; 4 mM MgCl_2_; 5 mM DTT; Complete protease inhibitor (Roche, Indianapolis, IN)] with the antibody and protein A-agarose beads (Roche) overnight at 4 °C. The co-immunoprecipitated RNA fraction was purified by phenol extraction. Alternatively, the protein fraction from immunoprecipitated material and total extract was obtained and analyzed by western blot, using the AGO1 antibody at a 1:1000 dilution.

### Plasmid constructs and generation of composite transgenic *hairy roots*

The Pvu-miR1514a precursor (202 bp) was amplified from genomic DNA and cloned into the pENTR/SD/D-TOPO vector (Invitrogen). To obtain a miR1514a over-expression construct, we used the Gateway LR Clonase in a recombination reaction (Gateway system, Invitrogen) between pENTR-premiR1514a and pBA-DC-TdT. This expression plasmid contains the 35S CaMV promoter, and the tdTomato gene (red-fluorescent protein) as a visible reporter gene (Naya *et al.*, 2014). To obtain the miR1514a STTM construct we followed the Short Tandem Target Mimicry strategy (Yan *et al.*, 2012). The resulting DNA fragment (113 bp) was cloned into the pENTR-SD vector (Invitrogen). The insert in this plasmid (pENTR-STTM-1514a) was then recombined into pBA-DC-TdT to obtain pBA-STTM-miR1514a-TdT. All constructs were confirmed by DNA sequencing (Unidad de Síntesis y Secuenciación de ADN, IBT-UNAM).

The empty pBA-DC-TdT vector and the resulting pBA-pre-miR1514a-TdT (OE-1514a) and pBA-STTM-miR1514a-TdT (STTM-1514a) plasmids were introduced into *Agrobacterium rhizogenes* K599 strain, and used for plant transformation as described previously (Estrada-Navarrete *et al.*, 2007) with minor modifications, in three independent experiments with 15 plants each. Plants were maintained for *hairy root* formation for 2 weeks and we obtained 2–6 independent TdT fluorescence positive roots per plant, which were pooled in each independent experiment and kept at –70 °C until further use.

### RNA-seq experiments and gene ontology analysis of common bean transgenic *hairy roots*

Common bean transgenic *hairy roots* carrying the appropriate constructs (EV, OE-1514a, or STTM-1514a) from two independent sets of samples were collected and processed to obtain total RNA as described above. Total RNA (1 μg) from each of the six samples was assessed for quality and integrity using a Bioanalyzer 2100 (Agilent Technologies, Santa Clara, CA), processed to obtain cDNA libraries using the True-Seq stranded mRNA Library Prep kit (Illumina, San Diego, CA), and sequenced using a NextSeq 500 platform (Illumina) with a configuration of 150 cycles for 2 × 75 paired-end sequences (Unidad Universitaria de Secuenciación Masiva de DNA, IBT-UNAM). After removing low-quality reads, all libraries contained between 32 and 47 million total reads of which 23 to 33 million reads per library mapped to the *P. vulgaris* ver. 1.0 genome available at Phytozome (www.phytozome.net/commonbean.php). Differential expression analysis was obtained using reads in the fastq format mapped to the *P. vulgaris* reference transcript compilation PhytozomeV10_Pvulgaris_218_v1.0.transcript.fa using the Bowtie aligner (Langmead *et al.*, 2009), applying the parameters -aS -X 600 --offrate 1. The resulting alignment was normalized using the eXpress software (Roberts and Pachter, 2013) with the -rf-stranded parameter since the data were generated using a strand-specific dUTP method. The normalized counts were used as input for the differential expression analysis using the scripts included with the Trinity software selecting the EdgeR package (Haas *et al.*, 2013). RNA-seq data in the form of raw data for six samples (EV, OE-1514a, or STTM-1514a, with replicates) have been deposited in the NCBI database as a Bioproject (accession PRJNA342643).

To predict functional relationships among differentially expressed genes found in the RNA-seq experiments, the enriched gene ontology (GO) categories obtained by topGO (https://bioconductor.org/packages/release/bioc/html/topGO.html;*P*-value <0.05) were classified based on their parental GO terms using the CateGOrizer tool (http://www.animalgenome.org/tools/catego/) with the ‘consolidated single occurrences count’ option. The final count of secondary categories for the corresponding parental categories was represented in a graph using the ggplot2 package in R (http://ggplot2.org/).

### In silico *DNA motif discovery analysis*

To identify DNA sequence motifs we used the MEME discovery tool with default parameters (Bailey *et al.*, 2009). We retrieved a region of 1000 nt upstream from selected genes using sequence data available for the *P. vulgaris* genome (www.phytozome.net/commonbean.php;Goodstein *et al.*, 2012).

To discover sequence motifs present in NAC transcripts in *P. vulgaris* and other legume species using the MEME discovery tool, a sequence fragment starting from the miRNA recognition sequence and ending at the stop codon was retrieved from different NAC TF sequences from the common bean genome, and homologous genes from *Glycine max* and *Medicago truncatula* were also retrieved using sequence data available from annotated genomes (www.phytozome.net;Goodstein *et al.*, 2012).

### Target prediction for NAC-derived phasiRNAs

To predict potential regulatory target mRNAs for the NAC-derived phasiRNAs, we used psRNATarget: A Plant Small RNA Target Analysis Server, (http://plantgrn.noble.org/psRNATarget;Dai and Zhao, 2011), using the default parameters. Manual addition of the phasiRNAs sequences was used to search targets using the transcriptome dataset for *P. vulgaris* (www.phytozome.net/commonbean.php).

## Results

### miR1514a shows different accumulation levels in roots during drought in two cultivars with different drought tolerance

We have previously reported that miR1514a levels in common bean (Pvu-miR1514a) increased in seedlings exposed to different abiotic stress conditions, as determined by northern blot assays (Arenas-Huertero *et al.*, 2009), and that its sequence is identical to that of *Glycine max* miR1514a (Subramanian *et al.*, 2008; Song *et al.*, 2011). The soybean genome encodes two *MIR1514* loci (*MIR1514a* and *MIR1514b*), which produce mature miRNAs that are identical in sequence except for a single nucleotide change towards the 3′-end (miRBase, version 21) (Subramanian *et al.*, 2008; Goettel *et al.*, 2014). We identified two loci encoding for *MIR1514a* (Chr. 3) and *MIR1514b* (Chr. 7) in the common bean genome, producing two mature 22-nt long miRNAs identical in sequence to Gma-miR1514a, and originating from asymmetrical precursors (see Supplementary Fig. S1A–C). We confirmed that both loci are expressed under the conditions tested in this study (Supplementary Fig. S1D, E). Because both common bean loci produce a miRNA identical in sequence to Gma-miR1514a, for the purposes of this study we will we not distinguish between them and we will address both common bean miRNAs as miR1514a.

**Fig. 1.**
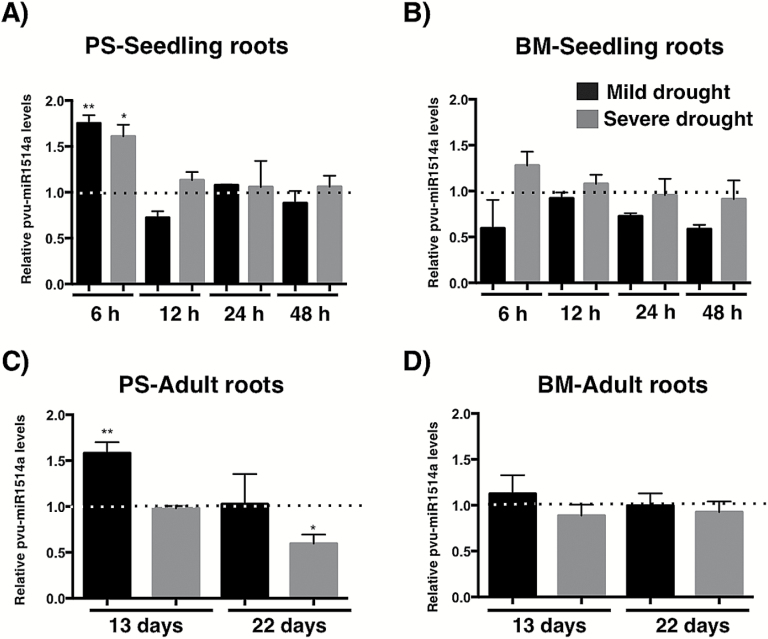
miR1514a shows differential accumulation levels in roots of two common bean cultivars with different drought tolerance. Northern blot analysis of Pvu-miR1514a accumulation levels in (A) Pinto Saltillo (PS) seedling roots, (B) Bayo Madero (BM) seedling roots, (C) PS adult plant roots, and (D) BM adult plant roots. Seedlings were subjected to mild drought by irrigating with 1/4 of field capacity (fc), while adult plants were irrigated with 1/2 fc. Severe drought was imposed on seedlings by watering with 1/8 fc whereas adult plants were irrigated with 1/4 fc (See Methods for details). Dotted lines represent the Pvu-miR1514a levels for untreated samples at each time point. All Pvu-miR1514a values were normalized to the endogenous levels of U6 snRNA. Asterisks indicate a significant difference compared to the control conditions, as determined by Tukey’s multiple comparisons test (*P<0.05, **P<0.01), *n*=3.

To continue characterizing the response to stress of miR1514a, we exposed 5-d-old seedlings to the addition of ABA or to different stress factors, including high NaCl or oxidative stress induced by the herbicide Paraquat, and determined miR1514a levels by northern blot analysis (see Supplementary Fig. S2). In these initial experiments, we observed that the different treatments did not show changes in miR1514a accumulation. Thus, we focused on drought stress and we compared two common bean cultivars, Pinto Saltillo (PS, drought-tolerant) and Bayo Madero (BM, drought-sensitive), known to have contrasting drought tolerance phenotypes as defined by their grain yield under terminal drought conditions (Acosta-Gallegos *et al.*, 1999; Terán and Singh, 2002). We determined the accumulation levels of miR1514a by northern blot analysis in seedlings subjected to mild or severe drought regimes for 6, 12, 24, or 48 h and we analyzed roots and hypocotyls of each cultivar (Fig. 1 and Supplementary Fig. S3). The results showed that miR1514a levels increased in PS roots and hypocotyls upon 6 h of treatment (Fig. 1A and Supplementary Fig. S3A), whereas in BM roots no changes were detected (Fig. 1B and Supplementary Fig. S3B).

Furthermore, we analyzed leaves and roots of PS and BM adult plants subjected to drought stress, where we found similar accumulation profiles to those observed for seedlings: namely, that miR1514a showed a significant increase in accumulation under drought in PS roots (Fig. 1C) but not in leaves (Supplementary Fig. S4A) when compared to well-irrigated conditions, while BM plants showed no significant difference in roots (Fig. 1D) or leaves (Supplementary Fig. S4B).

### miR1514a targets the mRNA of a NAC transcription factor under drought

**Fig. 2.**
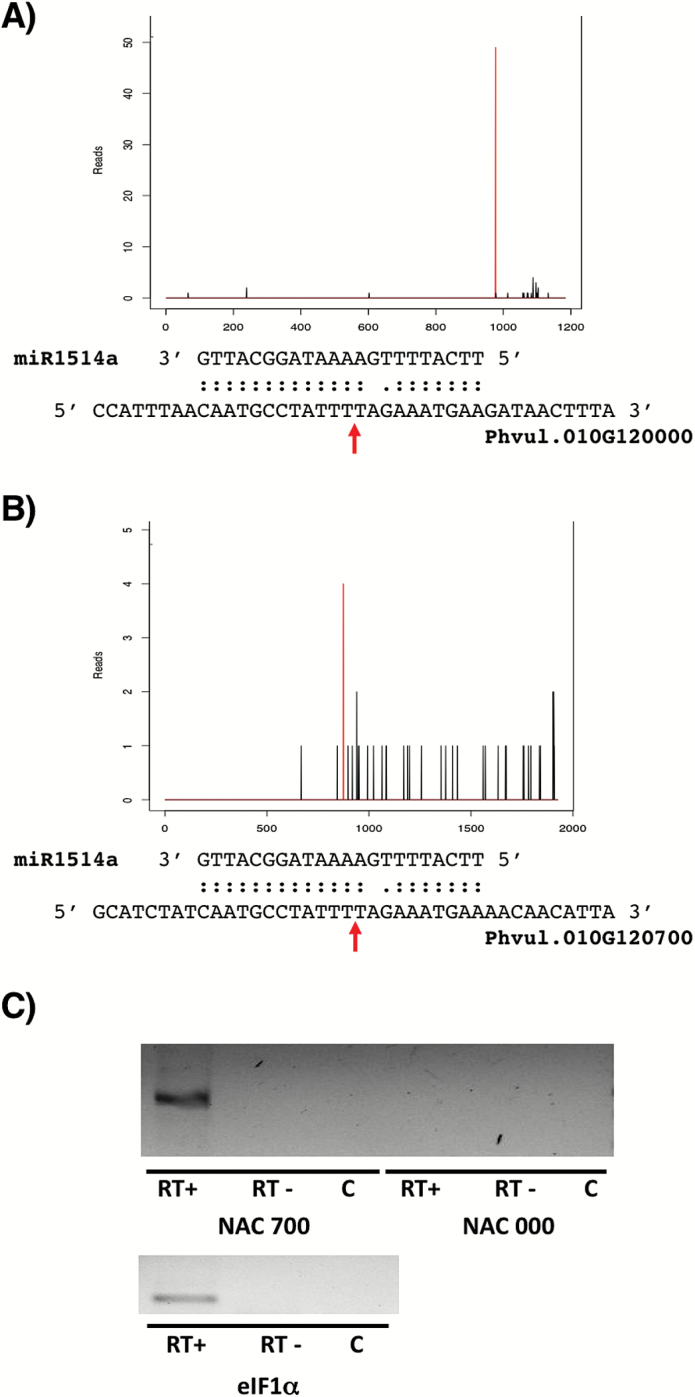
miR1514a targets the transcripts of two different NAC-TFs. (A) Phvul.010G121000 (NAC 000) and (B) Phvul.010G120700 (NAC 700) are targeted by miR1514a. The peak values show the cleavage site defined by Degradome analysis (Formey *et al.*, 2015). (C) RT-PCR assays showing the presence of NAC 700 and NAC 000 transcripts in roots of adult plants. RT+ and RT– refer to the presence or absence of reverse transcriptase enzymes in the cDNA reaction; C is the PCR negative control. NAC 700 shows an amplicon of 360 bp, and the NAC 000 amplicon has a length of 248 bp. Amplification of the eIF4 transcript was used as positive control in cDNA reactions. (This figure is available in colour at *JXB* online.)

Recently, we reported the genome-wide identification of miRNA targets in *P. vulgaris* by employing a Degradome or PARE analysis from seedling samples (Formey *et al.*, 2015), where miR1514a was found to target two transcripts encoding transcription factors of the NAC family, named Phvul.010G121000 (or NAC 000) and Phvul.010G120700 (or NAC 700) (Fig. 2A, B). This result was consistent with previous reports in *G. max* describing the mRNA targets of Gma-miR1514a as NAC TF-encoding transcripts (Arikit *et al.*, 2014). In contrast to the degradome results obtained from seedlings, only NAC 700 transcript was detected in roots from adult plants (Fig. 2C; Formey *et al.*, 2015).

**Fig. 3.**
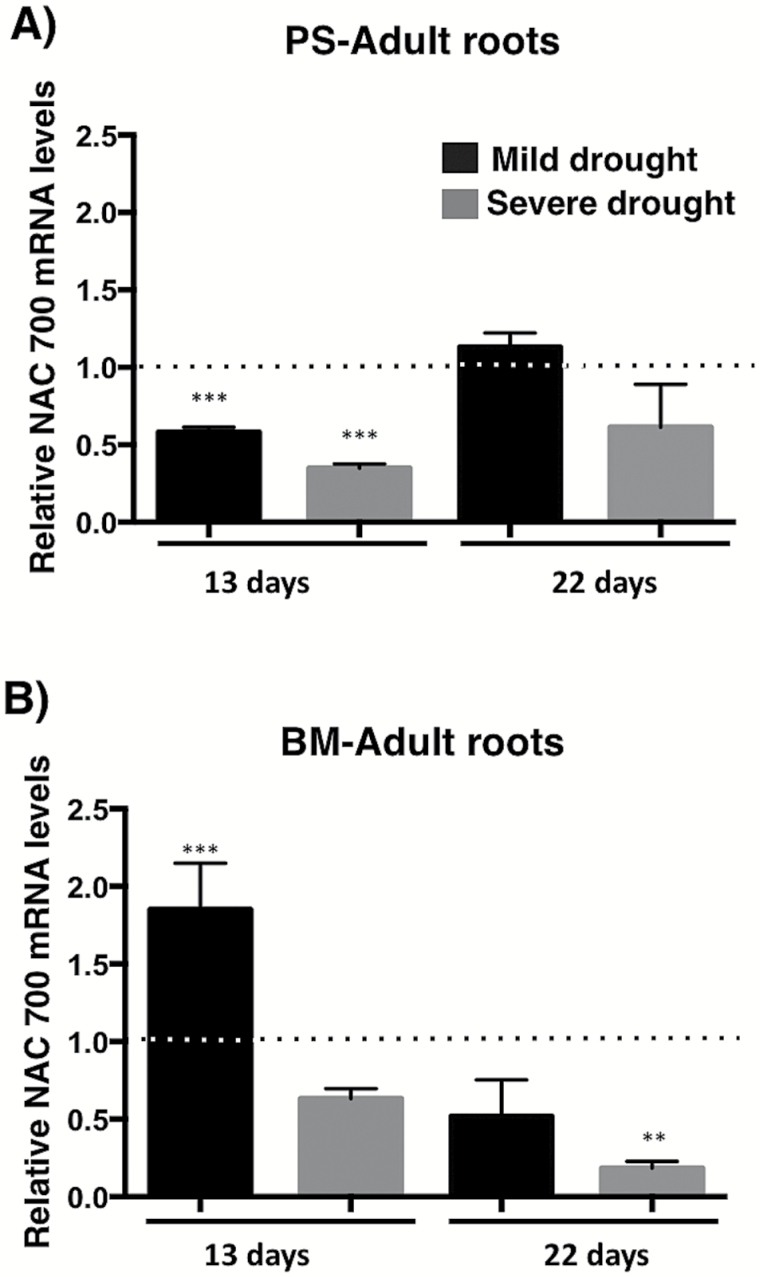
The drought resistant Pinto Saltillo (PS) cultivar shows reduced NAC 700 levels under drought conditions. (A) Levels of NAC 700 mRNA in roots of PS adult plants determined by RT-qPCR. (B) Levels of NAC 700 mRNA in roots of Bayo Madero (BM) adult plants determined by RT-qPCR. Mild drought represents irrigation to 1/2 field capacity (fc). Severe drought is 1/4 fc. Samples were collected at 13 or 22 d after water deprivation. Expression of NAC 700 was normalized with Skp16 mRNA levels. Asterisks indicate a significant difference compared to control conditions, as determined by Tukey’s multiple comparisons test (**P<0.01, ***P<0.001), *n*=3.

To confirm these data, a time-course experiment was performed in adult plants of PS and BM cultivars that were subjected to mild and severe drought treatments, and RT-qPCR analysis was carried out using RNA from roots to determine NAC 700 transcript accumulation levels. In agreement with the miR1514a accumulation pattern (see Fig. 1), the results showed that NAC 700 transcript abundance decreased in PS roots from stressed plants (Fig. 3A), whereas no significant change was detected for this transcript in BM roots from drought-treated plants, except at 22 d upon severe drought, which might represent an effect unrelated to miR1514a regulation (Fig. 3B).

### NAC 700 mRNA is processed into phasiRNAs upon cleavage by miR1514a

**Fig. 4.**
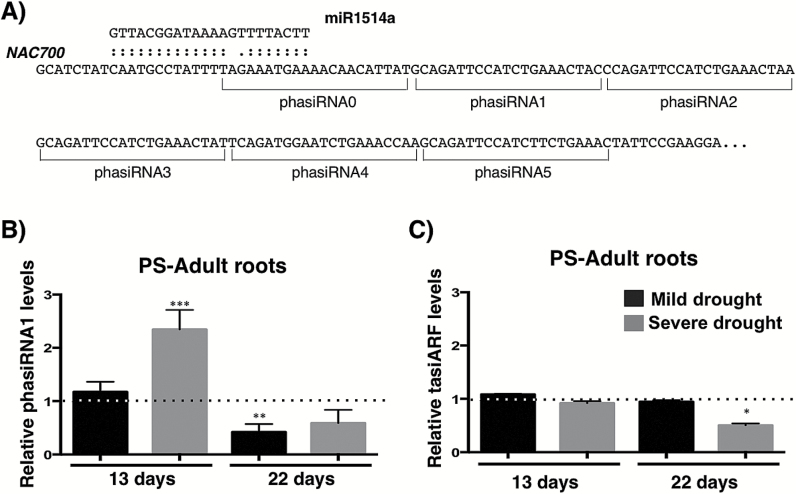
phasiRNA1 levels are increased during a drought time-course in PS roots. (A) Scheme of miR1514a targeting of NAC 700 and phasiRNAs produced, including the numbering scheme used. Root samples of Pinto Saltillo (PS) adult plants were collected after 13 or 22 d after water deprivation and total RNA was used for RT-qPCR detection of small RNAs. (B) phasiRNA1 accumulation levels, (C) tasiARF accumulation levels. Dotted lines represent the small RNA levels for untreated samples at each time point normalized to 1. Mild drought represents irrigation to 1/2 field capacity (fc) and severe drought is 1/4 fc. Accumulation levels of small RNAs were normalized to those of U6 snRNA. Asterisks indicate a significant difference compared to control conditions, as determined by Tukey’s multiple comparisons test (*P<0.05, **P<0.01, ***P<0.001), *n*=3.

Because miR1514a is 22 nt in length, we hypothesized that cleavage of its target mRNA would trigger the formation of phased small RNAs, or phasiRNAs (Chen *et al.*, 2007). Thus, considering the proposed miR1514a mRNA targets as candidates for phasiRNA formation, we applied a previously reported algorithm for phasiRNA loci identification (Chen *et al.*, 2007), which employed our previous sRNA-seq dataset (Pelaez *et al.*, 2012) to assign a *P*-value score. We found that NAC 700, but not NAC 000, showed a significant score (*P*=5.9 × 10^−6^ and ‘Not Detected’, respectively). As a control for this analysis we also recovered *TAS3* (*P*=9.1 × 10^−5^), a transcript that generates phasiRNAs upon miR390-mediated processing, known as tasiARFs (Axtell *et al.*, 2006) (see Supplementary Table S1). Moreover, we also found the NAC 700-derived phasiRNAs present in an independent sRNA-seq dataset of common bean (Nakano *et al.*, 2006; Pelaez *et al.*, 2012) (Supplementary Table S2). As part of our recent genome-wide analysis in common bean to uncover phasiRNAs-generating loci, we also identified the NAC 700 locus (Formey *et al.*, 2015) using an independent bioinformatical approach (Zhai *et al.*, 2011) (see Fig. 4A for a diagram).

To evaluate the production of NAC 700-derived phasiRNAs in common bean roots under drought conditions, we performed RT-qPCR analysis to determine their accumulation levels during drought time-course experiments where we had previously detected increased miR1514a levels and reduced NAC 700 levels (see Figs 1 and 3). The results indicated the presence of a subset of the phasiRNAs, which showed strikingly distinct accumulation levels (see Supplementary Fig. S5). Only one phasiRNA reproducibly increased during drought in PS roots, corresponding to the 21-mer RNA immediately following the first phasiRNA defined by miRNA cleavage, denoted hereafter as phasiRNA1 (Fig. 4A, B). In comparison, the accumulation levels of the unrelated tasiARFs derived from the *TAS3* transcript were unaffected under the same stress conditions (Fig. 4C).

### Modulation of miR1514a levels affects NAC 700 mRNA abundance

**Fig. 5.**
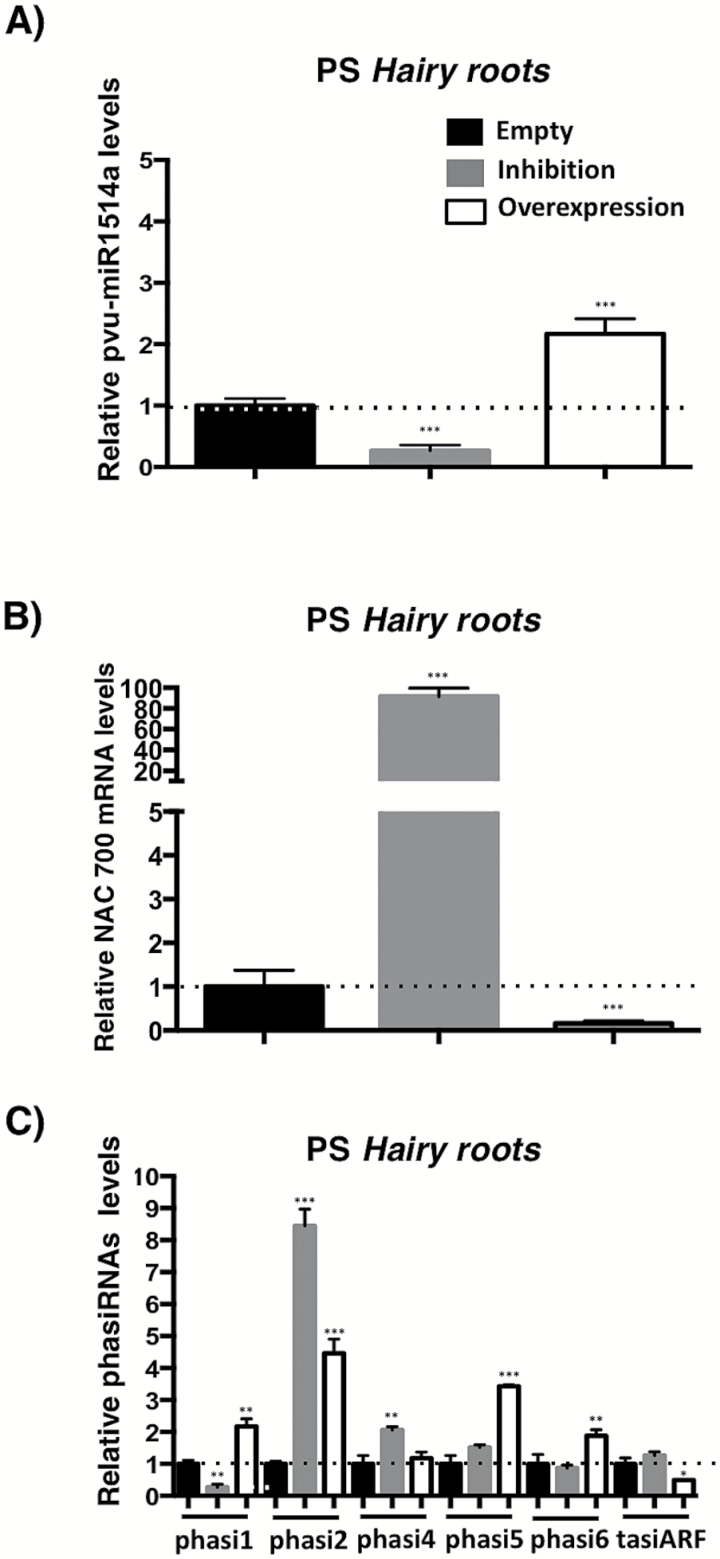
Inhibition and overexpression of miR1514a has an effect on NAC 700 transcript and phasiRNA1 levels. Transgenic *hairy roots* of Pinto Saltillo (PS) plants carrying the STTM-1514a construct, OE-1514a, or the empty vector were collected and total RNA was used for RT-qPCR assays to detect accumulation levels of (A) miR1514a, (B) NAC 700 transcript, and (C) NAC-derived phasiRNAs and tasiARFs. Accumulation levels of small RNAs were normalized to those of U6 snRNA and NAC 700 was normalized to Skp 16 mRNA. Dotted lines represent the RNA levels for untreated samples at each time point normalized to 1. Asterisks indicate a significant difference compared to control conditions, as determined by Tukey’s multiple comparisons test (*P<0.05, **P<0.01, ***P<0.001), *n*=3.

To provide further evidence of the direct role of miR1514a in regulating NAC 700 mRNA, we generated common bean transgenic *hairy roots* over-expressing the miR1514a precursor (OE-1514a) or transgenic *hairy roots* with reduced levels of active miR1514a using a Short Tandem Target Mimicry construct (STTM-1514a) aimed at inhibiting miRNA activity, as previously reported (Yan *et al.*, 2012). As expected, transgenic OE-1514a *hairy roots* showed an increased level of mature miR1514a, while the accumulation of NAC 700 mRNA was decreased. By contrast, we observed an increase in NAC 700 transcript levels when miR1514a was inhibited (Fig. 5A, B). Therefore, these results are consistent with a direct regulation of the NAC 700 transcript by miR1514a.

Next, we determined the accumulation of the NAC-derived phasiRNAs in these transgenic *hairy roots*. This analysis showed that even though not all individual phasiRNAs reached consistent accumulation profiles, both phasiRNA1 and phasiRNA6 showed reduced levels when miR1514a was inhibited, and increased levels when miR1514a was over-expressed (Fig. 5C, and see Fig. 4A for a diagram of phasiRNAs produced), consistent with their formation upon NAC 700 mRNA processing. In contrast, the levels of the unrelated tasiARFs under these conditions were largely unaffected (Fig. 5C).

The transgenic *hairy root* system successfully revealed the relationship between miR1514a and phasiRNA production. However, there is an extremely low efficiency in obtaining stable transgenic plants in common bean and this precluded our analysis of phenotypic traits caused by altered levels of miR1514a and/or NAC 700.

### NAC 700-derived phasiRNA1 associates with AGO1

**Fig. 6.**
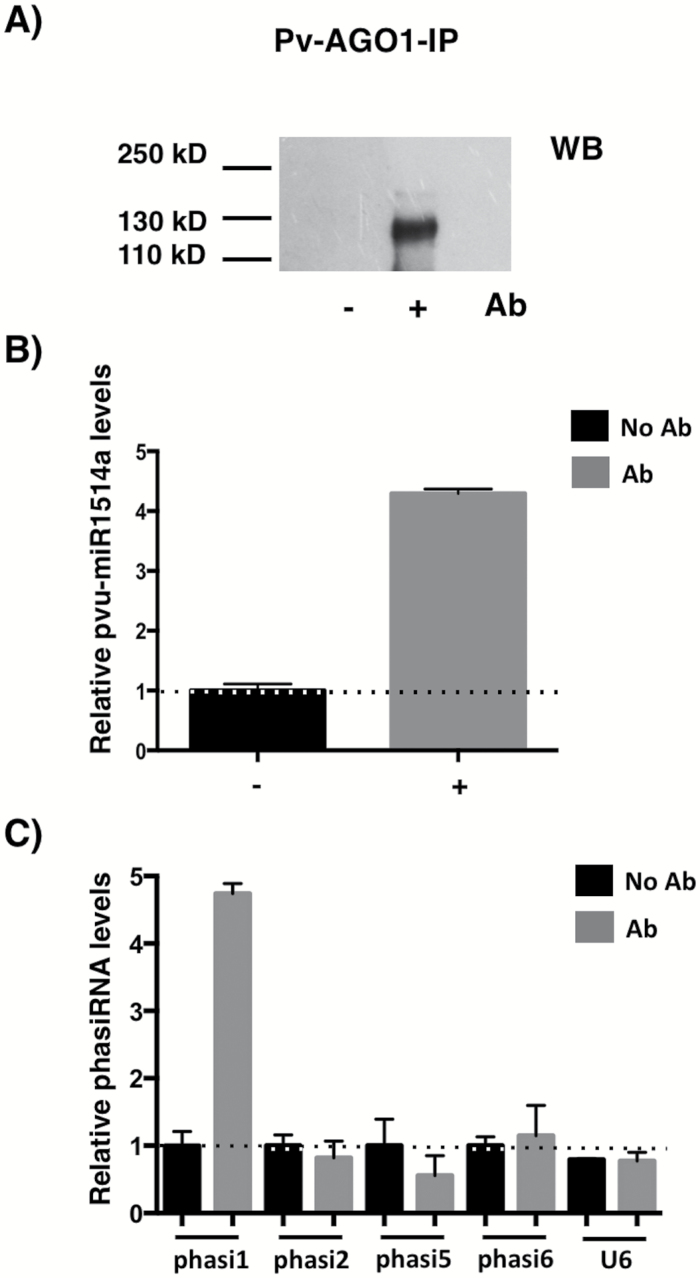
NAC 700-derived phasiRNAs associate with AGO1. Immunoprecipitation (IP) reactions were carried out using total protein extracts from Pinto Saltillo (PS) roots of plants subjected to drought and an antibody directed to the Pv-AGO1 protein. (A) Western blot showing AGO1 IP reactions with (+) or without (–) the AGO1 antibody. Total RNA from IP reactions was recovered and used to determine small RNAs by RT-qPCR: (B) levels of miR1514a, and (C) NAC-derived phasiRNAs and U6 snRNA in the absence or presence of the AGO1 antibody (Ab). Error bars represent the standard error of three technical replicates. Results from one representative experiment are shown.

To explore the potential functionality of the NAC-derived phasiRNAs we wanted to see whether they could be detected in association with AGO1-containing protein complexes. To that end, we performed immunoprecipitation (IP) experiments using an antibody raised against *P. vulgaris* AGO1 (see Methods). For this, total cell protein extracts were obtained from roots of PS adult plants subjected to drought. We successfully recovered Pv-AGO1 as determined by western blots of the IP fractions, which showed enrichment for miR1514a, while U6 snRNA, an unrelated RNA, was not recovered in the IP fractions (Fig. 6A–C illustrates one of two independent experiments that showed similar patterns).

We then determined the presence of different NAC-derived phasiRNAs in AGO1 complexes. We observed that only phasiRNA1 was enriched in the IP fraction, while other NAC-derived phasiRNAs were not consistently recovered (Fig. 6C). These results indicate that phasiRNA1 is recruited into AGO1-containing protein complexes and suggest that it is functional in PTGS. However, other NAC-derived phasiRNAs that we did not recover could be associated to other AGO protein complexes, or their low abundance could limit our ability to detect them reliably.

### Genome-wide effects of modulating miR1514a activity

**Fig. 7.**
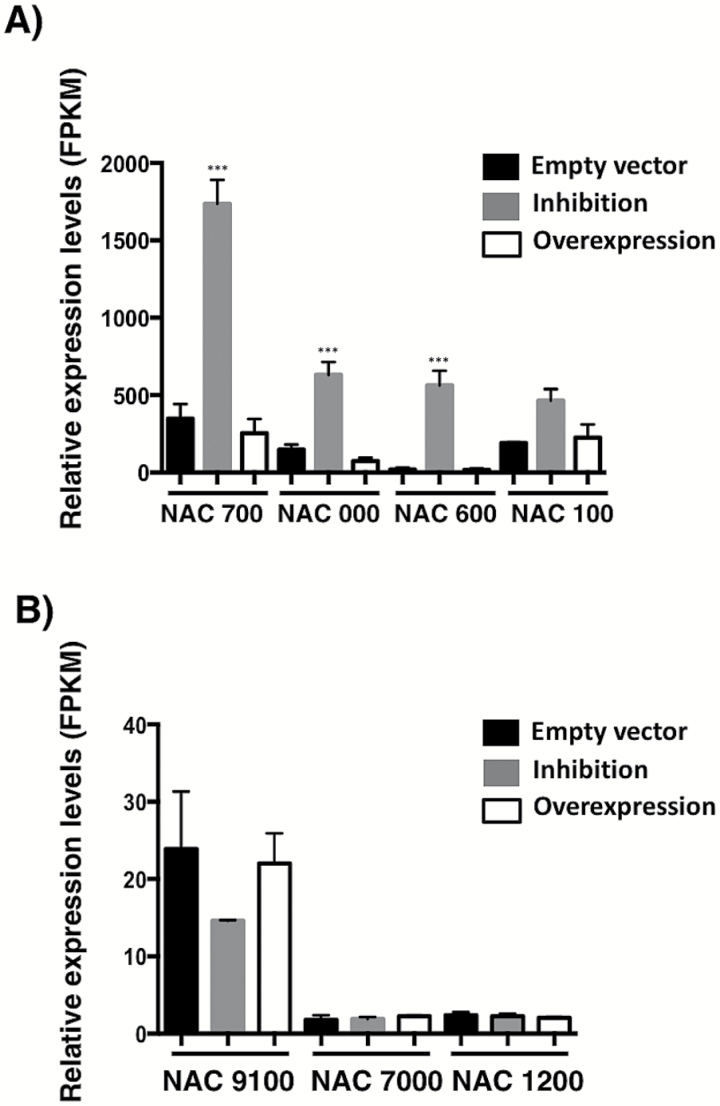
Transgenic *hairy roots* with reduced miR1514a levels reveal effects on NAC TF transcripts. Total RNA from two sets of transgenic *hairy roots* carrying the STTM-1514a construct, OE-1514a, or the empty vector, was obtained and analyzed by RNA-seq. (A) NAC 700 and other related NAC transcripts are modulated in roots carrying the miR1514a inhibition construct. (B) miR1514a has no effect on other unrelated NAC TFs lacking the miRNA recognition site. FPKM = fragments per kilobase of exon per million reads mapped. Asterisks indicate a significant difference compared to the empty vector samples, as determined by Tukey’s multiple comparisons test (***P<0.001).

**Fig. 8.**
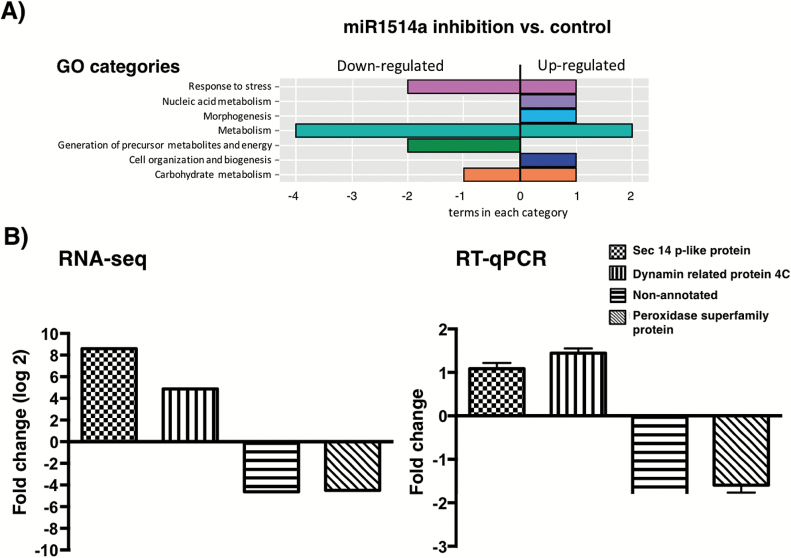
Genome-wide analysis of genes affected by inhibition of miR1514a. RNA-seq data was obtained from transgenic *hairy roots* carrying the STTM-1514a construct, OE-1514a, or the empty vector. (A) Gene ontology (GO) categories of mRNAs showing differential expression in roots carrying the miR1514a inhibition construct. (B) Comparison between RNA-seq data and RT-qPCR of selected genes with significant changes in miR1514a inhibition in *hairy roots*. (This figure is available in colour at *JXB* online.)

To explore the consequences of miR1514a activity in relation to gene expression, we generated composite plants for overexpression or inhibition of miR1514a (OE-1514a or STTM-1514a, respectively), or containing the empty vector (EV, used as a control). Total RNA samples from two independent sets of transgenic *hairy roots* were subjected to RNA-seq analysis. While we observed an increased accumulation of miR1514a in the OE-1514a roots (see Fig. 5A, and Supplementary Fig. S6), RNA-seq analysis of these samples revealed a very limited number of differentially expressed genes (five up-regulated and seven down-regulated genes, *P*-value =0.01, FDR=0.01, data not shown). Among these genes, we did not observe the expected reduced levels of NAC 700 mRNA; thus the miR1514a overexpression levels attained in these samples were not sufficient to produce a significant change and therefore these results were not analyzed further. In sharp contrast, we observed a large number of up- and down-regulated genes in roots where the miR1514a activity was inhibited (61 and 129 genes, respectively, *P*-value =0.01, FDR=0.01, Supplementary Table S3). Within this dataset we detected the corresponding up-regulation of NAC 700 mRNA but not of other unrelated NAC TFs (Fig. 7A and 7B, respectively). Thus, we determined significantly enriched gene ontology (GO) categories for the up- and down-regulated genes (see Supplementary Table S3). Based on Biological Process, in the up-regulated genes these categories included four metabolism terms, biogenesis, and morphogenesis (Fig. 8A), and included two HSP70 homologs and one *SHAVEN* 3 (SHV3) homolog involved in glycerol metabolism and important for root hair elongation (Hayashi *et al.*, 2008). For down-regulated genes, we again detected metabolism-related terms and responses to stress, including four different peroxidases, and a homolog of the PYR1-like ABA receptors (Kline *et al.*, 2010). We then analyzed the enriched GO terms based on Molecular Function, where we observed an enrichment for nucleic acid binding activity in the up-regulated genes, which contained two DCL homologs, two RDR homologs, and a HEN1 homolog. In the case of down-regulated genes, terms such as protein binding and antioxidant activity were recovered. To evaluate the significance of the results obtained in the RNA-seq experiments using a different approach, we selected up- and down-regulated genes (based on their fold-change values) to detect them by RT-qPCR in the RNA samples employed for high-throughput sequencing. Consistently, four of the selected genes showed a similar trend of accumulation to that observed in the transcriptome analysis (Fig. 8B).

Finally, we reasoned that if phasiRNAs act in PTGS, inhibition of miR1514a in transgenic *hairy roots* should result in reduced phasiRNA activity. To explore this idea, we first predicted regulatory targets of phasiRNAs using the psRNATarget server (see Supplementary Table S4). However, based on the RNA-seq data we did not find changes in these transcripts upon miR1514a deregulation (Supplementary Fig. S7), suggesting that phasiRNAs do not act *in trans*. Interestingly, other NAC transcripts related to NAC 700 showed a significant increase in their accumulation when miR1514a was inhibited (see Fig. 7A), suggesting they are targeted by miR1514a and/or by NAC-derived phasiRNAs in transgenic *hairy roots*.

## Discussion

In contrast to Arabidopsis and other model legume species, little is known about the post-transcriptional regulation in common bean during drought and even less is known about the contribution of miRNAs and other small RNAs, despite the great importance of gaining knowledge about this crop for improvement for food production to face population and climate changes. Therefore, in this study we aimed to characterize the activity of the common bean miR1514a during water deficit conditions.

### miR1514a shows differential expression levels in response to drought in common bean

In an attempt to contribute to our knowledge of miRNAs in common bean, in 2009 Arenas-Huertero and co-workers reported cloning of 16 conserved and six non-conserved miRNAs, including miR1514a which showed expression levels modulated by low water availability conditions (Arenas-Huertero *et al.*, 2009). Here, we analyzed the miR1514a expression levels using two common bean cultivars with contrasting drought tolerance (Terán and Singh, 2002; Rosales-Serna *et al.*, 2004; Sanchez-Valdez *et al.*, 2004; Padilla-Ramirez *et al.*, 2007; González *et al.*, 2008). Our results indicate that the terminal drought-resistant cultivar PS has a specific pattern of miR1514a modulation at two different developmental stages that was not detected in the susceptible BM cultivar, suggesting that it may mediate downstream events leading to stress responses.

### miR1514a targets a NAC TF mRNA for degradation in roots

A recent genome-wide analysis to identify miRNA targets showed two NAC transcription factor transcripts for miR1514a, Phvul.010G120700 (NAC 700) and Phvul.010G121000 (NAC 000) (Formey *et al.*, 2015). Similarly, a homologous NAC TF mRNA is targeted by miR1514a in soybean, supporting these findings (Arikit *et al.*, 2014). Here, we focused our attention on NAC 700 because its mRNA accumulation was decreased when miR1514a was up-regulated in roots of adult plants subjected to water deficit, while NAC 000 was not detected. These observations were consistent with the results obtained from transgenic *hairy roots*, where miR1514a accumulation levels were modulated, and showed an inverse effect on NAC 700 mRNA abundance, hence indicating that miR1514a directly targets NAC 700 mRNA. Nevertheless, we do not discard the possibility that with different growth conditions, different cultivars, and/or at other developmental stages, NAC 000 – or other related NAC TF mRNAs – might be expressed and subjected to miRNA regulation.

In plants, several miRNAs regulate mRNAs encoding transcription factors (Jones-Rhoades *et al.*, 2006), indicating that miRNAs can have an impact in miRNA–TF gene expression regulatory networks (Osella *et al.*, 2011; Megraw *et al.*, 2016). In addition, miRNAs can act in cell–cell or organ–organ communication (Megraw *et al.*, 2016), and they can even work at long distances from where they are produced, being transported through phloem (Buhtz *et al.*, 2008; Lin *et al.*, 2008; Pant *et al.*, 2008), thus effectively amplifying their regulatory effects. Upon cleavage of an initial transcript, production of secondary small RNAs may represent another way of signal amplification, as could be the case for phasiRNAs.

### miR1514a-directed cleavage of NAC 700 mRNA triggers phasiRNA formation

In *A*. *thaliana,* tasiRNAs have been widely studied, being produced mainly from four *TAS* genes; miR173 regulates *TAS1a/b/c* and *TAS2* transcripts, while miR390 and miR828 target *TAS3* and *TAS4*, respectively (Axtell *et al.*, 2006; Fei *et al.*, 2013). With the advent of high-throughput sequencing of small RNAs, phasiRNA identification has been developed and more *PHAS* loci have been annotated, as is the case for *TAS5*, *TAS6* (Arif *et al.*, 2012; Li *et al.*, 2012), and many *TAS*-like (*TASL*) loci (Zhai *et al.*, 2011; Xia *et al.*, 2013). In comparison, phasiRNAs have not been widely studied with the exception of a few crop plants, such as rice, maize, and soybean (Johnson *et al.*, 2009; Song *et al.*, 2012; Arikit *et al.*, 2014). In general, identification of phasiRNA loci has employed algorithms in genome-wide analyses (Chen *et al.*, 2007; Howell *et al.*, 2007), including *Phaseolus* (Formey *et al.*, 2015). In *G. max* miR1514a targets a NAC TF mRNA, subsequently triggering production of phasiRNAs (Arikit *et al.*, 2014). Likewise, in common bean we observed in sRNA-seq datasets that the NAC 700 locus is a source of phasiRNAs, consistent with its processing by miR1514a. Moreover, when the activity of miR1514a was modulated in transgenic *hairy roots* or during water deficit conditions, the accumulation of NAC 700-derived phasiRNAs was also affected, indicating that production of phasiRNAs triggered by the action of the 22-nt-long miR1514a on a NAC TF transcript is conserved in *P. vulgaris*, and possibly other legumes as well.

### The role of miR1514a during water deficit responses

**Fig. 9.**
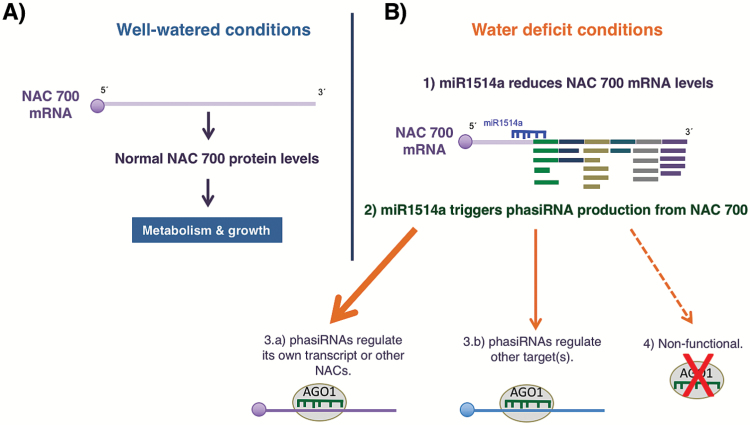
miR1514a amplifies RNA silencing by phasiRNA production through the cleavage of NAC 700 transcript during drought stress. This model summarizes our results. Under non-stress conditions, NAC 700 TF regulates downstream gene expression to contribute to normal metabolism and growth (A). Upon water limitation (B), miR1514a is induced and targets NAC 700 transcript for cleavage (1) and phasiRNA production (2). Some phasiRNAs may have their own regulatory targets such as NAC 700 and/or other NAC homologous transcripts, as discussed in the text (3.a) or other unrelated mRNAs (3.b). Alternatively, they may be non-functional (4).

The activity of miR1514a on the transcript of NAC 700 has two outcomes. Firstly, it reduces the levels of the NAC 700 transcription factor, thus affecting downstream gene expression; secondly, miR1514a action results in phasiRNA production, in particular phasiRNA1, which could have its own regulatory roles, including amplification of the response initiated by miR1514a. Either one or both outcomes could be directly involved in stress responses and we will discuss them next (Fig. 9).

### *NAC-derived phasiRNAs could target other transcripts for cleavage* in trans

NAC-derived phasiRNAs may have two alternative outcomes: (1) phasiRNAs may target other transcripts *in trans*, or (2) phasiRNAs may not be functional (shown as 3.a and 3.b, or 4, in Fig. 9). Remarkably, the sequence of NAC 700-derived phasiRNA1 is conserved in different common bean NAC TF transcripts and is present in related NAC TF genes from other legume species (see Supplementary Fig. S8), suggesting that it is functional, although conservation due to other factors, including NAC 700 mRNA amino acid coding capacity, can not be ruled out. Our results can be compared to miR390, which directs the processing of *TAS3* in different plant species to generate phasiRNAs where only a subset of these, known as tasiARFs, are conserved and regulate the transcripts of ARF2, ARF3/ETT, and ARF4 *in trans* (Axtell *et al.*, 2006; Fei *et al.*, 2013*),* suggesting that phasiRNA1 acts as a tasiRNA. Accordingly, we found that phasiRNA1 is recruited into AGO1 complexes under water deficit conditions in adult plant roots; hence, together with its conservation, this result suggests that phasiRNA1 acts *in trans* and might target a mRNA to perform a particular function in the Fabaceae family. The phasiRNA1 contains a G at the 5′’-end, which differs from the U preference known for AGO1 (Mi *et al.*, 2008), although other conserved miRNAs such as miR172 also contain a G at the 5′-end, indicating that this is not an absolute prerequisite for association with AGO1. However, in the transcriptome analysis of transgenic *hairy roots* with reduced miR1514a activity we found no changes in potential transcripts regulated by phasiRNAs. In contrast, the levels of other related NAC TF mRNAs were altered, suggesting that phasiRNAs and/or miR1514a are directly involved in their modulation *in trans* (shown as a thick arrow in 3.a in Fig. 9). The potential activity of NAC-derived phasiRNAs on NAC transcripts could be indicating a new tasiRNA activity on their own transcripts, a hypothesis that remains to be tested.

### Potential regulatory targets of the NAC 700 transcription factor

To evaluate the molecular consequences of miR1514a activity, we performed a transcriptome analysis of transgenic *hairy roots* focusing on the transcription factor NAC 700. From the differentially expressed genes we determined enriched GO terms indicating that genes related to metabolism, biogenesis, morphogenesis, and responses to stress were affected when we modulated miR1514a abundance. In *A. thaliana* several NAC TFs have been associated with drought-stress, including NAC016, NAC019, NAP, NAC053/NTL4, NAC055, NAC072, and NAC096 (Tran *et al.*, 2004; Lee and Park, 2012; Zhang and Gan, 2012; Xu *et al.*, 2013), indicating the relevance of this TF family in stress responses. The differentially expressed genes obtained by RNA-seq analyses could be a direct consequence of NAC 700 TF activity. If so, the regulatory regions of these genes might contain common sequence motifs, potentially representing a NAC 700 binding site. However, we failed to uncover any significant sequence motifs, probably due to the presence of direct and indirect gene expression changes occuring in the transgenic *hairy roots*.

Finally, among the differentially expressed genes defined by the RNA-seq of transgenic *hairy roots* we identified a homolog of Sec14p (Phvul.006G137200, Fig. 8B), a protein initially defined as involved in vesicle trafficking in yeast (Bankaitis *et al.*, 1989). This mRNA was also differentially accumulated in response to water deficit in the roots of drought-resistant PS plants but not in the drought-sensitive BM plant roots (data not shown). This finding suggests that it is possible to identify gene expression changes related to miR1514a regulation that are reflected in gene expression changes caused by water deficit in PS and BM cultivars. It remains to be determined if other genes that were not considered in this analysis could also be affected by NAC 700 accumulation levels and water deficit conditions.

### Conclusions

Taken together, the results presented here illustrate the participation of the miR1514a regulatory module during water deficit conditions in *P. vulgaris*. It is possible that common bean and other legumes may have particular strategies for gene regulation based on other miRNAs and phasiRNA products to regulate stress responses. The recent description of the common bean genome (Schmutz *et al.*, 2014; Vlasova *et al.*, 2016) will provide invaluable knowledge for future PTGS studies. In addition, the different molecular participants in the miR1514a pathway could be used to guide crop improvement programs directed at the generation of new and more resistant cultivars of common bean and other legumes.

## Supplementary data

Supplementary data are available at *JXB* online

Figure S1. Pre-miR1514a is present in the common bean variety Pinto Saltillo.

Figure S2. Accumulation levels of miR1514a in hypocotyls and roots of common bean under different abiotic stresses.

Figure S3. miR1514a levels in hypocotyls of the Pinto Saltillo and Bayo Madero cultivars during water deficit.

Figure S4. miR1514a levels in leaves of adult plants of Pinto Saltillo and Bayo Madero cultivars during water deficit.

Figure S5. Some NAC-derived phasiRNAs in roots from adult plants have different levels during a drought time-course experiment.

Figure S6. Expression levels of RNA-seq control genes.

Figure S7. NAC-derived phasiRNA predicted targets are not affected in RNA-seq data.

Figure S8. PhasiRNA1 is conserved in common bean and others legumes.

Table S1. Probability of generating phasiRNAs for NAC genes.

Table S2. phasiRNAs reads derived from NAC 700 in two independent sRNA-seq experiments.

Table S3. Differential gene expression in STTM-1514a transgenic *hairy* roots.

Table S4. NAC-derived phasiRNA targets predicted by psRNATarget.

Table S5. Primers used in this study.

## Supplementary Material

Supplementary_figures_S1_S8

supplementary_tables_S1_S5
