# Supplementary material for: The legume miR1514a modulates a NAC transcription factor transcript to trigger phasiRNA formation in response to drought
Source: J Exp Bot. 2016 Oct 7;68(8):2013–26. doi: 10.1093/jxb/erw380 (PMC5429018; doi:10.1093/jxb/erw380)
Supplement: Supplementary_figures_S1_S8 [file erw380_suppl_Supplementary_figures_S1_S8.pdf]

Figure S1

A)

```
Pvu-miR1514a/b    UUCAUUUUGAAAAUAGGCAUUG
Gma-miR1514a      UUCAUUUUUAAAAUAGGCAUUG
Gma-miR1514b      UUCAUUUUUAAAAUAGACAUUG
Mtr-miR1514a      UUCAUUUCUAAAAUAGGCAUUG
Consensus:        *******  ******* *****
```

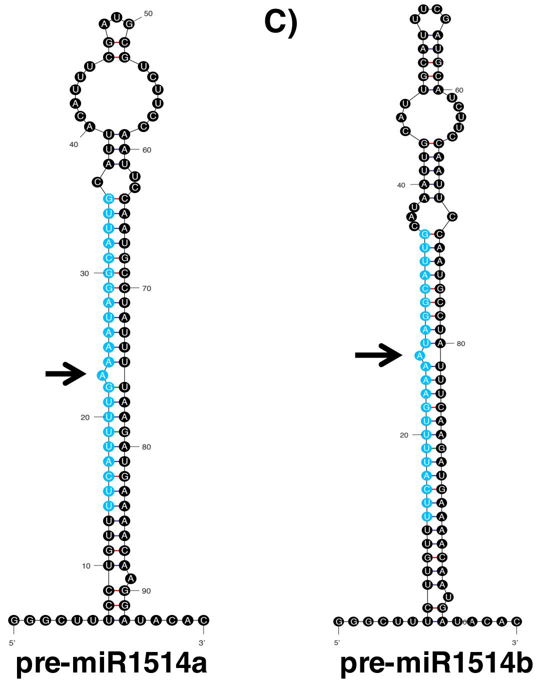

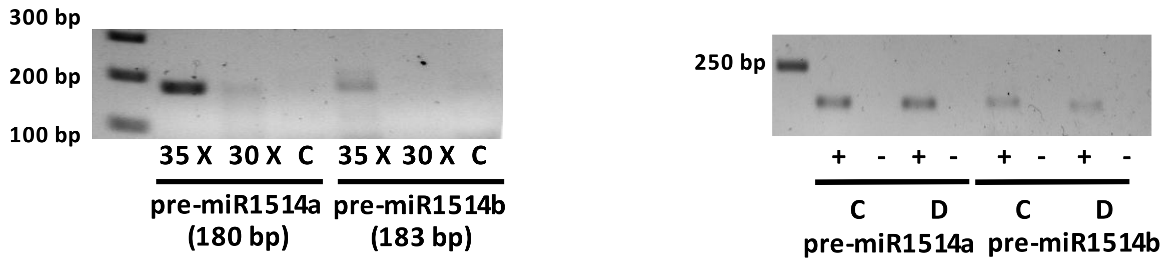

Figure S1: pre-miR1514a is present in common bean variety Pinto Saltillo. A) Sequence alignment of pvu-miR1514a/b to the sequence of *Glycine max* miR1514a and miR1514b (taken from miRBase: www.mirbase.org ver. 21 and http://mpss.udel.edu) and we have found and corroborated expression of a highly related small RNA in *Medicago truncatula* (Mtr-miR1514a, PS Romero and JL Reyes, unpublished results). Base substitutions are indicated by a grey square along the sequence. B) the structure of pre-miR1514a has a dG = -30.70 kcal/mol and C) pre-miR1514b shows a dG= -39.10 kcal/mol. Sequence of mature miRNAs is shown in light blue circles. Arrows indicate a mismatch position for the asymmetric precursor forms. Pre-miR1415a is located in Chr03 (pos. 23530620-23530821) and pre-miR1514b is situated in Chr07 (pos. 457103-457228). D) pre-miR1514a and pre-miR1514b in genomic DNA amplification at 35 and 30 cycles of PCR, respectively and E) pre-miR1514a and pre-miR1514b are present in cDNA amplification in two control (labeled as C) and drought (labeled as D) conditions. The '+' and '-' signs refer to presence of reverse transcriptase in the reaction.

Figure S2

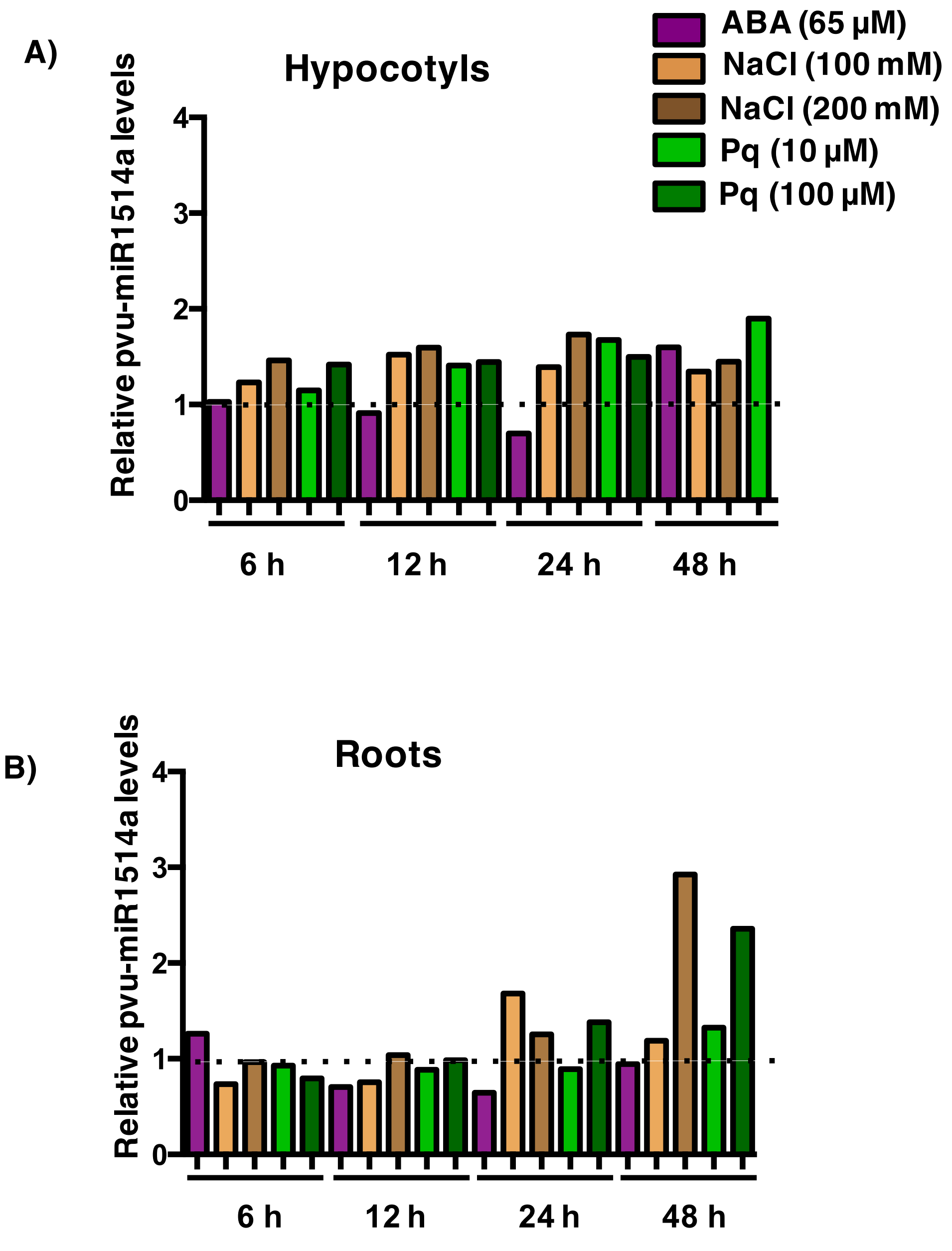

Figure S2: Accumulation levels of Pvu-miR1514a in hypocotyls and roots of common bean under different abiotic stresses. A) Northern blot analysis of hypocotyls from Pinto Saltillo seedlings, B) Northern blot analysis of roots from Pinto Saltillo seedlings. Pq= Paraquat. Dotted lines represent the Pvu-miR1514a levels for untreated samples at each time point. All Pvu-miR1514a values were normalized to the endogenous levels of U6 snRNA.

Figure S3

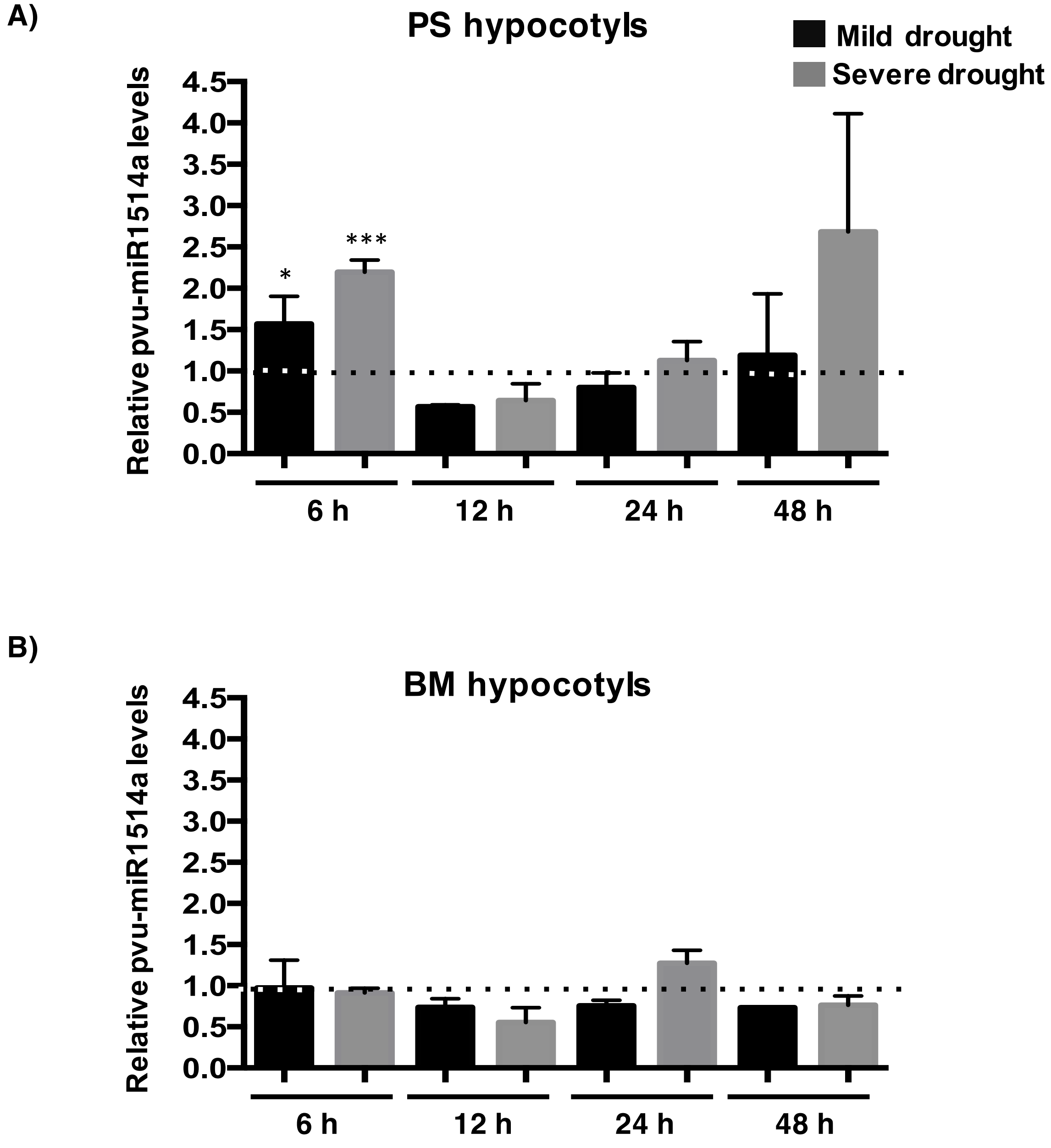

Figure S3: Pvu-miR1514a levels in hypocotyls of Pinto Saltillo (PS) and Bayo Madero (BM) cultivars during water deficit. A) Northern blot analysis of PS hypocotyls, B) Northern blot analysis of BM hypocotyls. Dotted lines represent the Pvu-miR1514a levels for untreated samples at each time point. Mild and severe drought, indicate drought treatments with 1/4 or 1/8 of field capacity watering, respectively. All Pvu-miR1514a values were normalized to the endogenous levels of U6 snRNA. Asterisks indicate a significant difference compared to the control conditions, as determined by Tukey's multiple comparisons test (*P< 0.05, **P<0.01, ***P<0.001), n = 3.

Figure S4

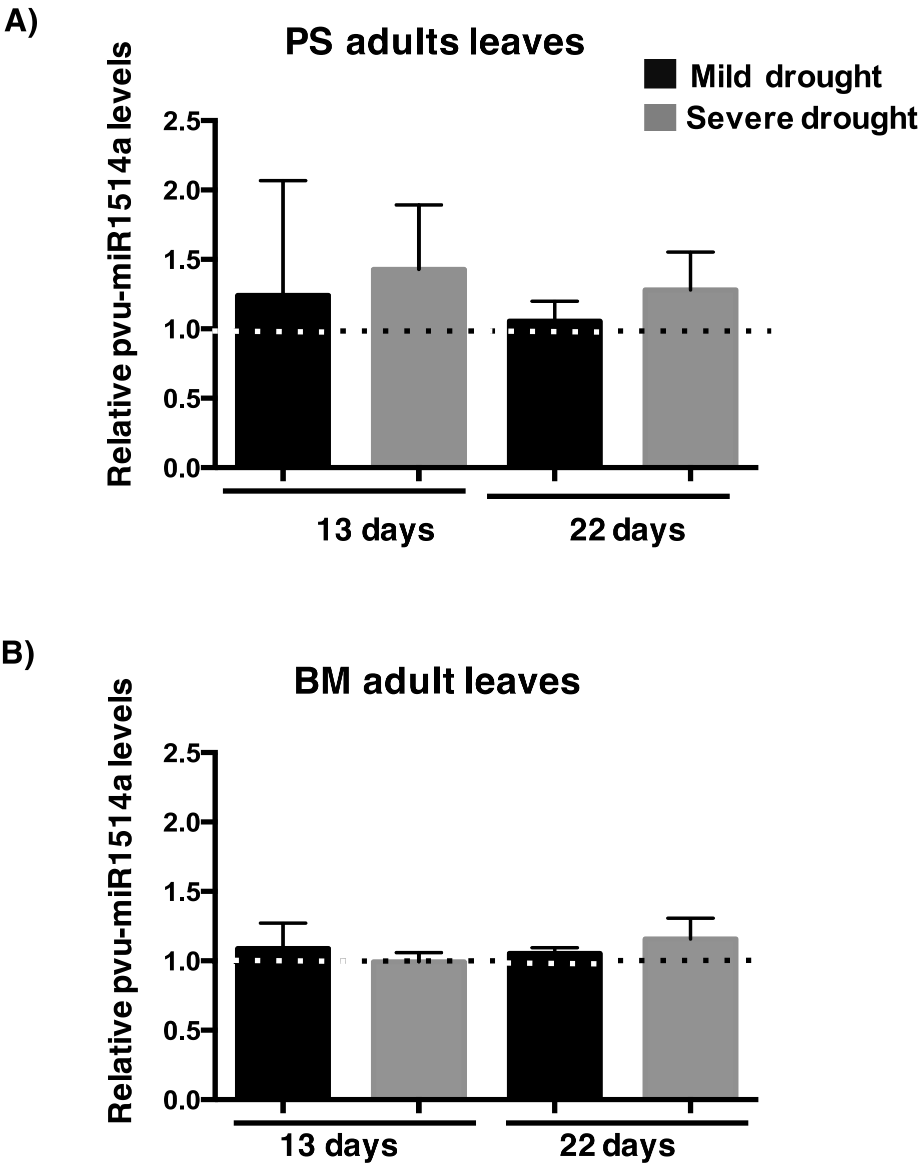

Figure S4: Pvu-miR1514a levels in adult leaves of Pinto Saltillo (PS) and Bayo Madero (BM) cultivars during water deficit. A) Northern blot analysis of PS leaves, B) Northern blot analysis of BM leaves. Mild drought represents irrigation to 1/2 of field capacity (fc) and Severe drought is 1/4 fc. Dotted lines represent the Pvu-miR1514a levels for untreated samples at each time point. All Pvu-miR1514a values were normalized to the endogenous levels of U6 snRNA. Asterisks indicate a significant difference compared to the control conditions, as determined by Tukey's multiple comparisons test (*P< 0.05, **P<0.01, ***P<0.001), n = 3.

Figure S5

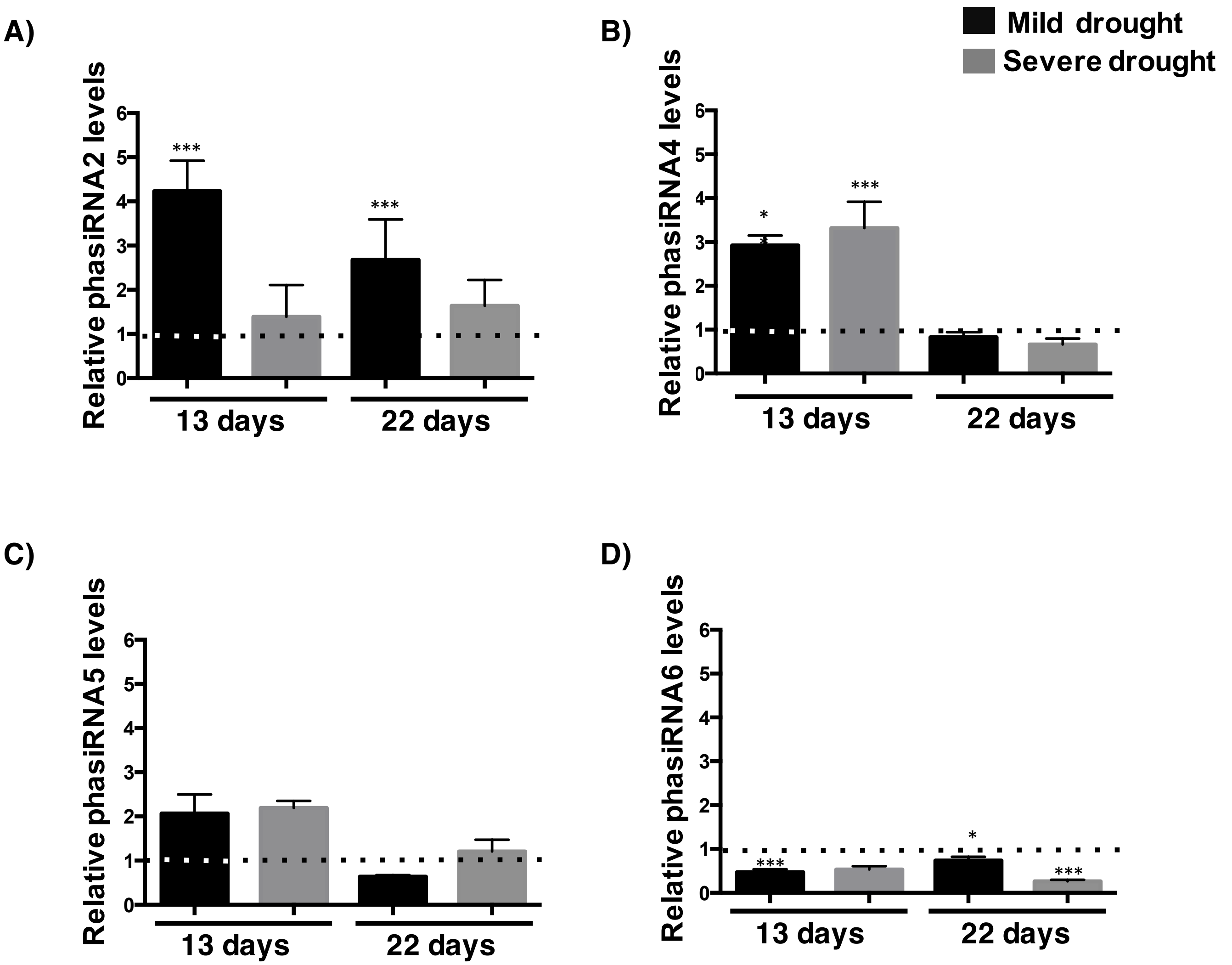

Figure S5: Some NAC derived phasiRNAs in adult roots have different levels during a drought time-course experiment. RT-qPCR analysis of phasiRNAs in PS leaves. Mild drought represents irrigation to 1/2 of field capacity (fc) and Severe drought is 1/4 fc. A) phasiRNA2, B) phasiRNA4, C) phasiRNA5, D) phasiRNA6. All phasiRNA values were normalized to the endogenous levels of U6 snRNA. Asterisks indicate a significant difference compared to the control conditions, as determined by Tukey's multiple comparisons test (*P< 0.05, **P<0.01, ***P<0.001), n= 3.

Figure S6

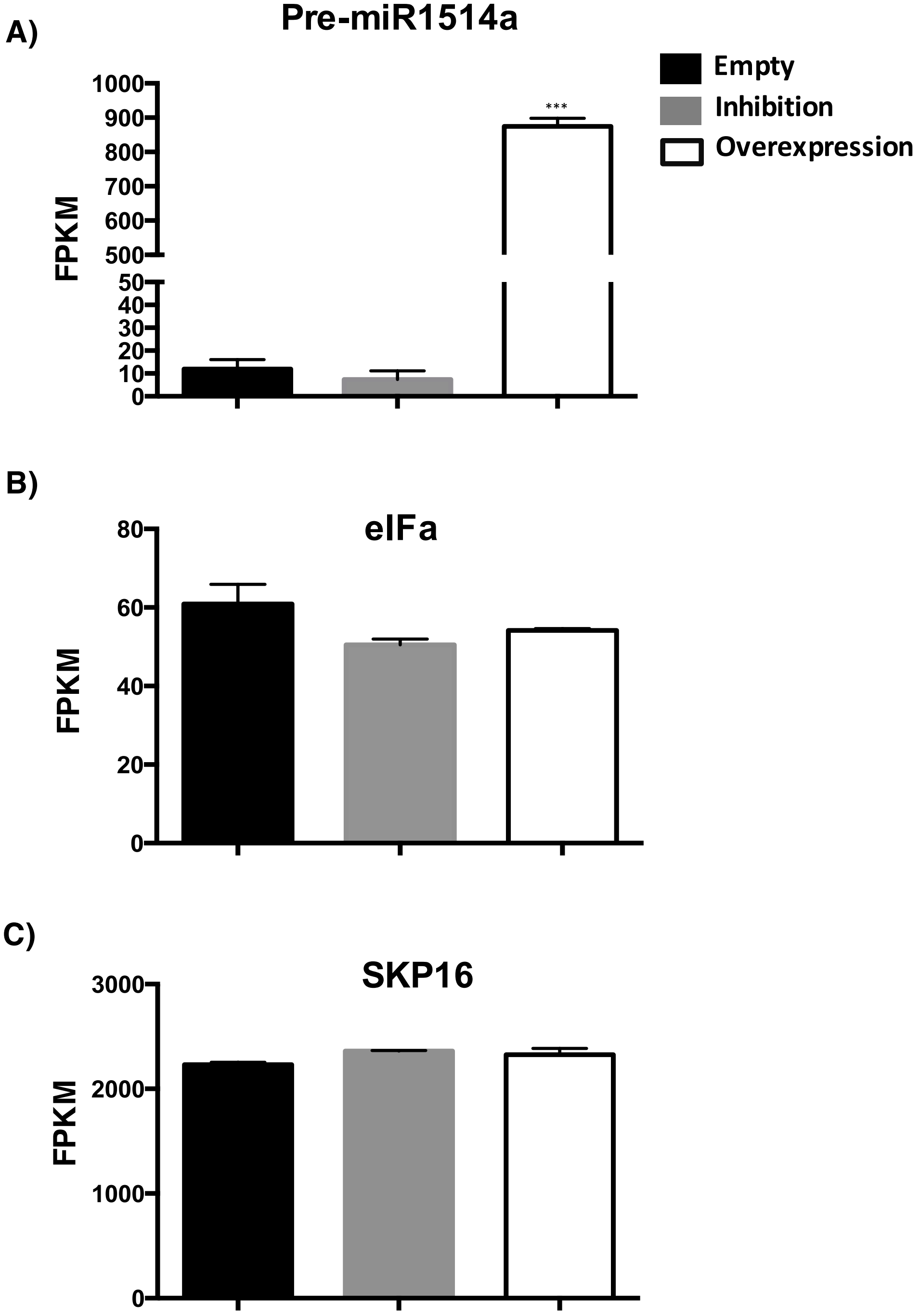

Figure S6: RNA-seq control genes. Expression levels of different genes found in RNA-seq datasets for transgenic hairy roots carrying the empty vector (black box, empty), STTM-1514a construct (gray box, Inhibition) or OE-1514a construct (white box, Overexpression). A) Accumulation levels of Pre-miR1514a, B) accumulation levels of RT-qPCR reference gene Phvul.004G075100 (eIF1a), and C) accumulation levels of RT-qPCR reference gene Phvul.011G053400 (SKP16). Asterisks indicate a significant difference compared whit the control conditions, as determined by Tukey's multiple comparisons test (*P< 0.05, **P<0.01, ***P<0.001). n= 2.

Figure S7

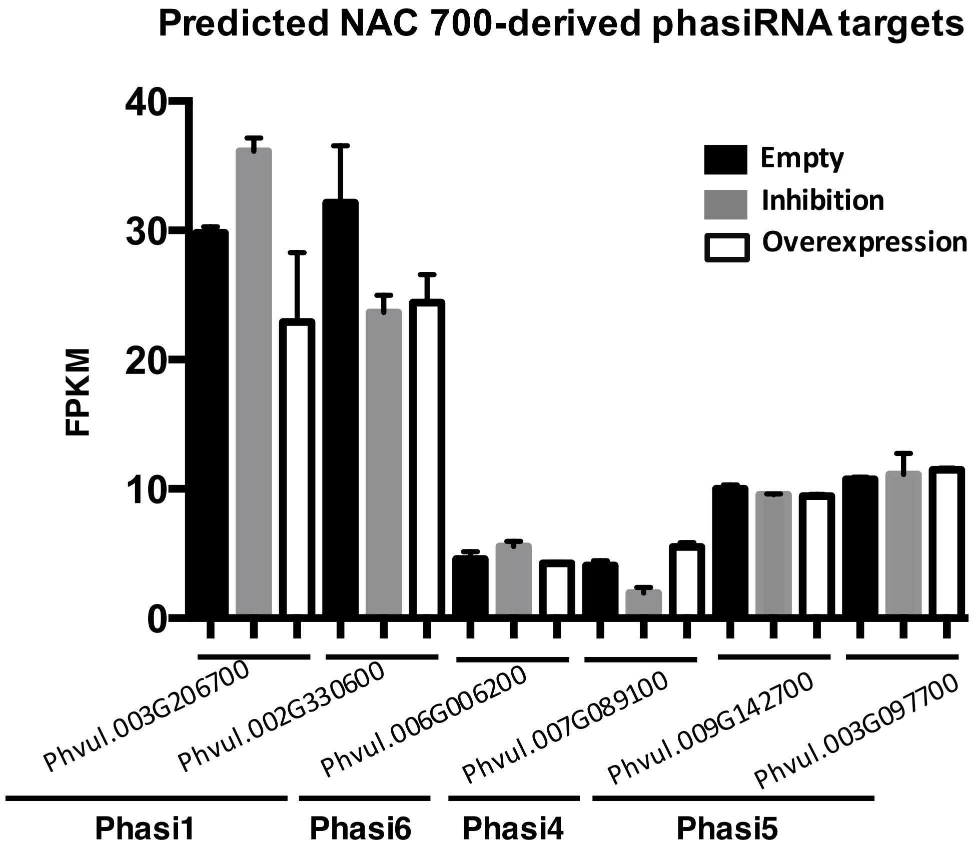

Figure S7: NAC-derived phasiRNA predicted targets are not affected in RNA-seq data. Potential targets for phasiRNAs were predicted using the psRNATarget tool. Expression levels of predicted target genes found in RNA-seq datasets for transgenic hairy roots carrying the empty vector (black box, empty), STTM-1514a construct (Inhibition, grey box) or OE-1514a construct (white box, Overexpression) are shown. Asterisks indicate a significant difference compared to the control conditions, as determined by Tukey's multiple comparisons test (*P< 0.05, **P<0.01, ***P<0.001), n=2.

Figure S8

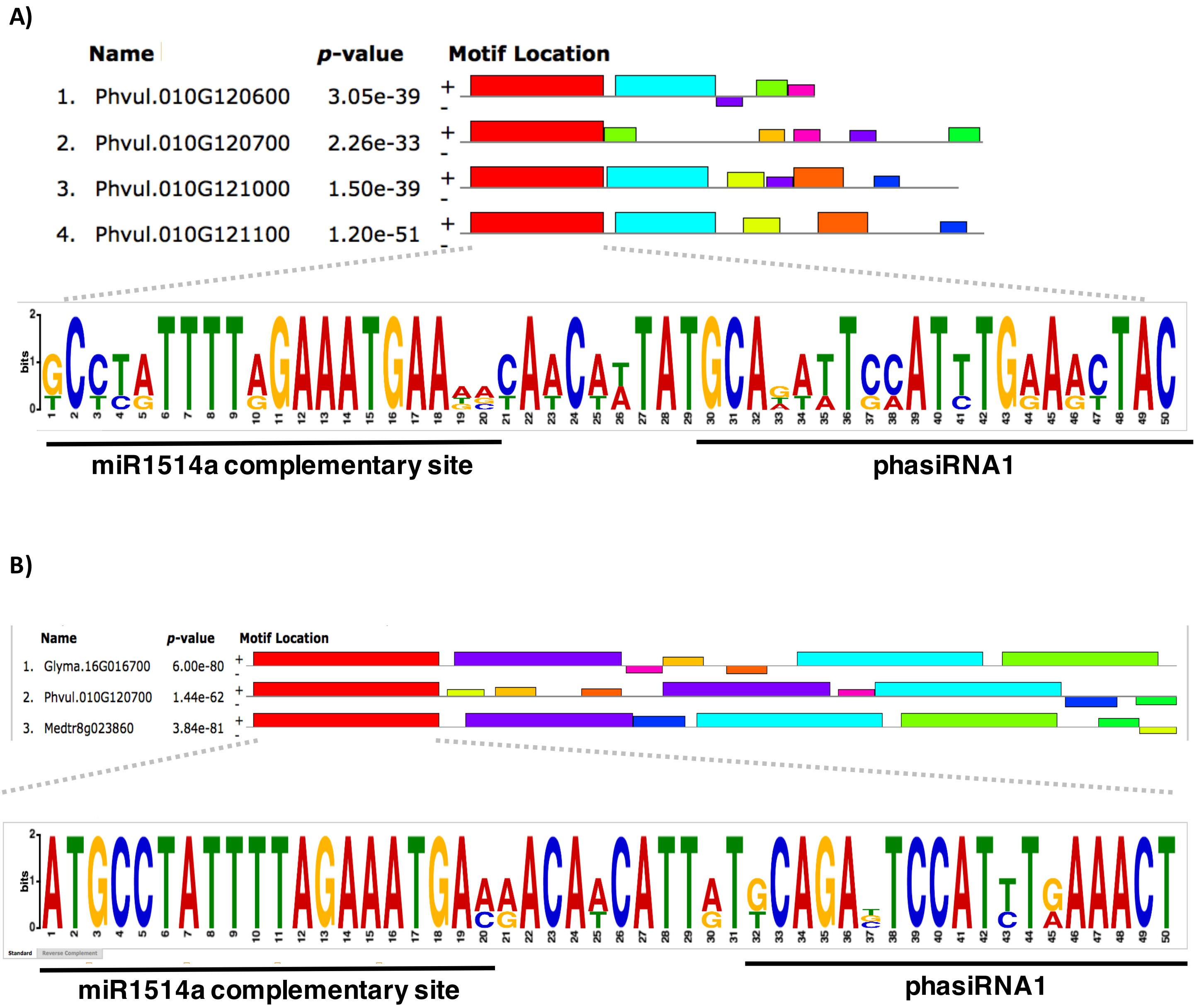

Figure S8: phasiRNA1 is conserved in common bean and other legumes. A) NAC TFs (Phvul010G120600, Phvul010G120700, Phvul010G121000 Phvul010G121100) in common bean contain a shared miR1514a complementary sequence and the phasiRNA1 sequence. B) miR1514a complementary sequence and phasiRNA1 are present in other miR1514-targeted NAC TF transcript in legumes. pvu = *Phaseolus vulgaris*, gma = *Glycine max* and mtr = *Medicago truncatula*.
